# Evolution of Multifaceted Sport-Related Concussion Management: A 25-Year Narrative Review of Multidomain Assessment and Multimodal Rehabilitation

**DOI:** 10.3390/sports14030112

**Published:** 2026-03-13

**Authors:** James Stavitz, Kenneth Swan, Adam Eckart, Thomas Koc, Jenna Tucker, Jennifer T. Gentile, Pragya Sharma Ghimire, Ryan Porcelli

**Affiliations:** 1 Department of Athletic Training Education, College of Health Professions and Human Services, Kean University, 1000 Morris Avenue, Union, NJ 07083, USA; 2 University Orthopaedic Associates, 2 Worlds Fair Dr, Somerset, NJ 08873, USA; 3 Department of Health and Human Performance, College of Health Professions and Human Services, Kean University, 1000 Morris Avenue, Union, NJ 07083, USA; 4 Department of Physical Therapy, College of Health Professions and Human Services, Kean University, 1000 Morris Avenue, Union, NJ 07083, USA

**Keywords:** sport-related concussion, concussion rehabilitation, multifaceted concussion management, multidomain assessment, multimodal rehabilitation, aerobic exercise therapy, vestibular rehabilitation, athletic trainers

## Abstract

Context: Sport-related concussion (SRC) management has evolved substantially over the past 25 years. Early paradigms emphasized prolonged physical and cognitive rest; however, growing evidence has demonstrated that recovery following SRC is multidimensional and influenced by interacting neurological, vestibular, autonomic, cervical, cognitive, and psychological systems. Consequently, contemporary clinical practice has shifted toward active, multifaceted rehabilitation approaches. Objective: We aimed to synthesize and contextualize the evidence supporting a multifaceted approach to sport-related concussion management from 2000 through 2025, with emphasis on implications for athletic training practice. Data Sources: A structured literature search was conducted using PubMed, SPORTDiscus, CINAHL, and Web of Science to identify peer-reviewed publications related to SRC evaluation, management, and rehabilitation. Study Selection: Studies published between 1 January 2000, and 31 December 2025 involving human participants with sport-related concussion or sport-like mechanisms of mild traumatic brain injury were included. Evidence from randomized controlled trials, cohort studies, systematic and narrative reviews, and major consensus or position statements was considered. Data Extraction: Relevant studies were reviewed and synthesized across key domains of SRC management, including aerobic exercise, vestibular and oculomotor rehabilitation, cervical spine management, multimodal and profile-based rehabilitation, return-to-learn strategies, psychological and behavioral health considerations, and implementation patterns within athletic training settings. Results: A total of 182 publications contributed evidence to one or more components of multifaceted SRC management. Across domains, evidence supports early, symptom-limited aerobic exercise; targeted vestibular and cervical rehabilitation; structured return-to-learn planning; and the integration of psychological support. Multimodal rehabilitation and profile-based clinical categorization approaches were associated with shorter recovery timelines and improved functional outcomes compared with rest-only strategies. Despite strong evidence, implementation variability persists across athletic training settings. Conclusions: Evidence accumulated over the past 25 years supports a shift toward active, individualized, and multidisciplinary approaches to SRC management. Athletic trainers are uniquely positioned to coordinate multifaceted care addressing the diverse contributors to concussion recovery.

## 1. Introduction

Sport-related concussion (SRC) remains one of the most common injuries managed by athletic trainers across all levels of sport. Epidemiological estimates suggest that approximately 1.1–1.9 million sport- and recreation-related concussions occur annually among U.S. youth, based on national surveillance data and emergency department–derived estimates from studies conducted in the mid-2010s [1]. However, true incidence is likely underestimated due to underreporting, variability in injury recognition, and differences in access to medical evaluation across sport settings. SRC incidence also varies by age, sex, and level of participation, with higher rates observed in adolescent and collegiate athletes participating in contact and collision sports.

Over the last 25 years, SRC has evolved substantially. Earlier models characterized concussion as a condition managed primarily through symptom monitoring and physical rest. It is now recognized as a complex, multidimensional injury involving neurological, vestibular, cervical, cognitive, and psychological components [2,3,4]. Early management strategies in the 2000s relied heavily on prolonged rest and the expectation that spontaneous neurological recovery would occur without targeted intervention [3,4]. However, growing evidence has demonstrated that strict rest may prolong recovery, exacerbate emotional symptoms, and delay return to normal functioning. As a result, clinical practice and consensus recommendations have shifted steadily. Contemporary management now emphasizes an active, multifaceted rehabilitation model [2].

### 1.1. Evolution of SRC Management Paradigms

This evolution has been driven by several key developments in research and practice. First, advancements in pathophysiological understanding have highlighted the interdependence of multiple systems in SRC recovery [2,3,4]. These include autonomic regulation, vestibular-ocular function, cervical spine mechanics, and psychological well-being [5]. Second, a series of prospective studies and randomized trials have supported the use of early, symptom-limited aerobic exercise as a therapeutic strategy. This evidence challenges long-standing rest-based paradigms [6]. Concurrently, vestibular and oculomotor rehabilitation has shown benefit for athletes presenting with dizziness, visual motion sensitivity, and balance impairments. Cervical spine assessment and treatment have also been prioritized as essential components in the management of post-concussive headache and dizziness [7].

### 1.2. Emergence of Multifaceted Rehabilitation Models

In parallel with the rise of active rehabilitation, a more structured and interprofessional approach to return-to-learn (RTL) and return-to-play (RTP) has emerged. Updated consensus statements, pediatric guidance, and athletic training practice documents now emphasize early, supported RTL progression and targeted academic accommodations. These documents also highlight the integration of mental health screening and referral pathways [2,8]. These developments collectively reinforce the importance of viewing concussion as more than a single-domain neurological event. Instead, concussion is recognized as an injury requiring coordinated, profile-based care across multiple physiological and psychosocial systems. Given their central role in injury evaluation, care coordination, and return-to-activity decision-making, athletic trainers are well positioned to operationalize this multifaceted model across clinical settings [3,9].

Despite this progression, variability persists in how SRC is evaluated and managed across athletic training settings. Implementation of multifaceted rehabilitation approaches differs widely based on resources, clinician training, and institutional protocols [3,10]. Moreover, research supporting individual rehabilitation modalities has expanded over time. However, fewer reviews have synthesized the evolution of these approaches across the full 25-year period during which SRC management has changed most dramatically [11,12].

### 1.3. Purpose and Scope of the Review

The purpose of this narrative review is to examine the evidence supporting a multifaceted approach to SRC management from 2000 through 2025, with specific emphasis on implications for athletic training practice. Rather than presenting a purely thematic synthesis, this review adopts a chronological and translational perspective. It traces how evolving evidence has progressively reshaped clinical practice over time. This review integrates findings across aerobic exercise, vestibular-ocular rehabilitation, cervical spine management, multimodal rehabilitation, RTL strategies, and psychological support, with RTP considered a key clinical outcome influenced by these rehabilitation domains [11,13]. By situating these domains within the historical evolution of concussion care and emphasizing their application to real-world athletic training settings, the review aims to bridge research evidence with contemporary clinical decision-making [11].

## 2. Methods

### 2.1. Study Design

This article is a narrative literature review designed to synthesize and contextualize the evolution of evidence supporting a multifaceted approach to SRC management. The review includes literature published between 1 January 2000, and 31 December 2025. A narrative review format was selected because SRC management encompasses multiple interdependent domains. These include physiological recovery, vestibular–ocular function, cervical spine mechanics, academic reintegration, psychological health, and physical rehabilitation, which extend beyond the scope of a single intervention or outcome suitable for systematic review or meta-analysis [14]. This approach also aligns with the overall goal of the review. Specifically, the review describes conceptual developments, summarizes evidence across rehabilitation domains, and integrates findings relevant to athletic trainers. Emphasis is placed on clinical translation and real-world applicability.

### 2.2. Search Strategy

A structured search of PubMed, SPORTDiscus, CINAHL, and Web of Science was conducted using combinations of terms related to concussion, rehabilitation, and multifaceted management [15]. Searches were conducted independently within each database using database-specific indexing terms and search strategies, with results reviewed collectively to identify relevant studies. Search terms included “sport-related concussion”, “mild traumatic brain injury,” “rehabilitation,” “active rehabilitation,” “aerobic exercise,” “vestibular therapy,” “oculomotor therapy,” “cervical spine,” “cervicogenic dizziness,” “multimodal physiotherapy,” “return-to-learn,” “return to school,” “academic accommodations,” “psychological factors,” “mental health,” and “persistent symptoms.” Searches were updated throughout manuscript development to make sure the inclusion of the most recent publications through late 2025. Reference lists from key clinical trials, systematic reviews, consensus statements, and professional position statements were also reviewed to identify additional relevant studies.

### 2.3. Eligibility Criteria

Studies were included if they were published in peer-reviewed journals between 2000 and 2025 and involved human participants with SRC or sport-like mechanisms of mild traumatic brain injury. Eligible studies addressed evaluation, management, or rehabilitation strategies relevant to contemporary multifaceted concussion care. Evidence from randomized controlled trials, cohort studies, and systematic reviews was considered. Narrative reviews introducing important conceptual frameworks were also included. Major consensus and position statements, including those from the Concussion in Sport Group, National Athletic Trainers’ Association, and American Academy of Pediatrics, were incorporated as well. Studies focusing solely on moderate or severe traumatic brain injury, biomechanics, or laboratory research without clinical translation were excluded. Diagnostic accuracy studies without management implications and non–peer-reviewed commentary were also excluded unless they represented formal practice recommendations.

### 2.4. Evidence Appraisal and Thematic Categorization

Because of the breadth and heterogeneity of the literature spanning 25 years, formal risk-of-bias scoring was not performed. This represents an inherent limitation of the narrative review design, as the absence of standardized quality appraisal introduces potential for selection bias and limits the ability to quantitatively compare study quality across the included evidence. Instead, studies were evaluated based on clinical relevance, methodological rigor, and contribution to understanding a specific rehabilitation domain. Strength of evidence was interpreted within the broader context of SRC management, with emphasis placed on consistency of findings across study designs, populations, and time periods [16].

When conflicting findings were identified, greater weight was assigned to higher-level evidence, including randomized controlled trials, systematic reviews, and consensus statements, while also considering the consistency and reproducibility of findings across studies. In cases where interpretation differed among authors, consensus was reached through iterative discussion to ensure alignment with the clinical objectives and scope of the review. No strict quantitative quality thresholds were applied; however, studies demonstrating clear methodological limitations or insufficient relevance to SRC management were deprioritized during synthesis.

Findings were organized into thematic domains representing the major components of multifaceted concussion care [17]. These included evolution of consensus recommendations, aerobic exercise and autonomic regulation, vestibular and oculomotor rehabilitation, and cervical spine assessment and management. Additional domains covered multimodal physiotherapy and profile-based care, RTL strategies, psychological and behavioral health considerations, and implementation patterns within athletic training settings. Study selection and thematic categorization were performed iteratively by the authors to make sure alignment with the clinical objectives of the review [14,17].

### 2.5. Narrative Synthesis Approach

**Figure 1:**
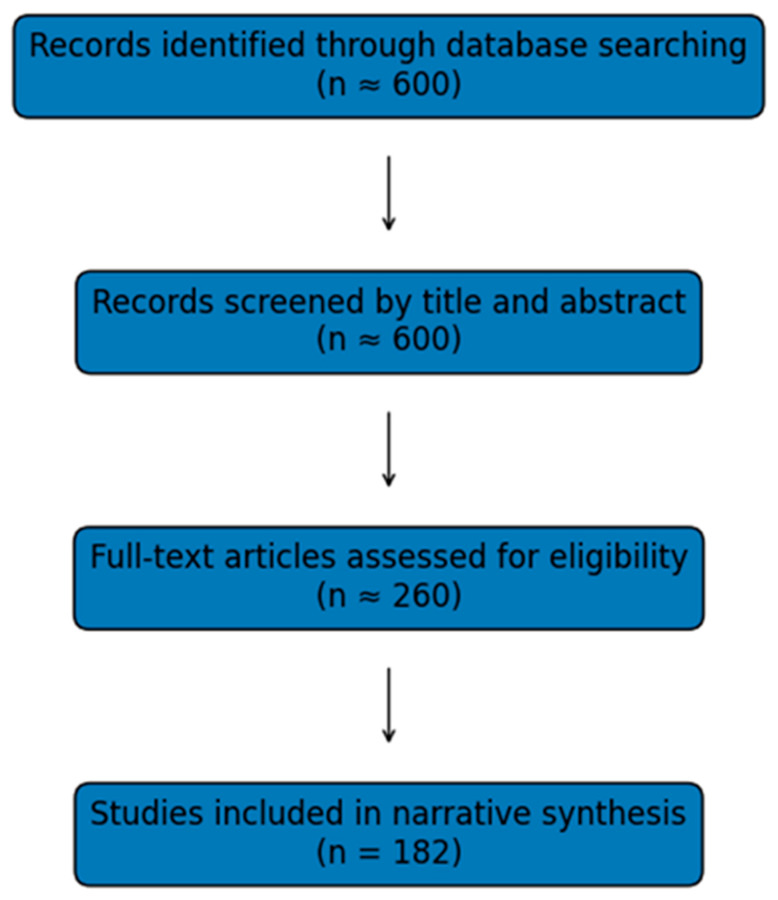
Literature Screening and Selection Flow Diagram. Records were identified through database searching across PubMed, SPORTDiscus, CINAHL, and Web of Science (n ≈ 600). After title and abstract screening, 260 full-text articles were assessed for eligibility. A total of 182 peer-reviewed publications met inclusion criteria and were incorporated into the narrative synthesis.

A structured narrative synthesis approach was deemed most appropriate for capturing the conceptual evolution and interprofessional nature of SRC management [17]. Many rehabilitation domains contain limited randomized controlled and randomized clinical trials. However, these areas include prospective studies, clinical frameworks, and emerging guideline recommendations that meaningfully influence practice but are not amenable to meta-analytic pooling. Unlike prior reviews that focus on individual rehabilitation domains, this narrative review synthesizes 25 years of evidence across all major components of multifaceted SRC management. These findings are situated within contemporary athletic training practices. The narrative format therefore allowed for integration of diverse evidence sources. This approach facilitated the development of a contemporary, multifaceted model of SRC management intended to guide athletic trainers and allied clinicians [14,15,16,17,18]. While this approach limits reproducibility compared with systematic review methods, it allows for integration of heterogeneous evidence and facilitates examination of the conceptual and clinical evolution of SRC management over time. The literature screening and study selection process is illustrated in Figure 1, which provides a visual summary of the narrative literature identification and screening process but does not represent a formal PRISMA systematic review workflow.

## 3. Results

### 3.1. Literature Search Yield

The literature search conducted across PubMed, SPORTDiscus, CINAHL, and Web of Science yielded more than 600 records published between 2000 and 2025 related to SRC. After title and abstract screening for relevance to concussion management and rehabilitation, approximately 260 full-text articles were reviewed. Of these, 182 peer-reviewed publications directly contributed evidence to one or more components of multifaceted SRC management. These studies formed the final body of literature synthesized in this review. The final body of evidence included 29 randomized controlled trials, 71 prospective cohort studies, 38 retrospective cohort or case-series studies, 24 systematic or scoping reviews, 12 narrative reviews introducing conceptual models, and 8 major consensus or position statements. The volume of rehabilitation-focused research increased substantially after 2010, reflecting a broader shift toward active concussion management approaches described in evolving international consensus statements [2,4]. More than 65% of included studies were published after 2015, reflecting a rapid expansion of evidence supporting active and multidomain concussion management described in contemporary consensus statements [2]. Included studies predominantly involved adolescent and collegiate athletes, with most evidence derived from these populations. In contrast, representation of professional and recreational sport populations was comparatively limited [1,2]. A domain-level synthesis of evidence supporting multifaceted sport-related concussion management is summarized in Table 1. Although aerobic exercise interventions have been supported by randomized controlled trials, evidence supporting other domains such as vestibular rehabilitation, cervical interventions, psychological support, and return-to-learn strategies has more commonly been derived from prospective cohort studies, observational investigations, and emerging clinical frameworks [2,4,9,14,15,16,17]. Reported percentage ranges and recovery timelines presented throughout the Results should be interpreted within the context of heterogeneous study designs, populations, and outcome measures across the included literature.

### 3.2. Evolution of Consensus and Guideline Recommendations

Between 2001 and 2023, six international concussion consensus statements were published by the Concussion in Sport Group. These statements represent a progressive shift in SRC management philosophy [2,19]. Key milestones in the evolution of sport-related concussion management and guideline development are summarized in Table 2. Early consensus statements emphasized symptom resolution and rest until asymptomatic, with minimal guidance regarding active intervention. The 2008 and 2012 Zurich consensus statements marked early recognition that prolonged strict rest might be detrimental, though formal rehabilitation strategies were not yet emphasized [20,21]. The 2016 Berlin consensus introduced explicit endorsement of early, symptom-limited physical activity following an initial rest period of 24 to 48 h [19]. The most recent 2022 Amsterdam consensus further advanced this paradigm by recommending early aerobic exercise, targeted vestibular and cervical rehabilitation, and individualized, profile-based care [2]. In parallel, professional organizations including the National Athletic Trainers’ Association and the American Academy of Pediatrics issued updated guidance reinforcing early RTL, multidisciplinary management, and avoidance of prolonged inactivity. These recommendations demonstrate convergence across international, pediatric, and athletic training-specific guidelines [9,22,23]. Collectively, these evolving consensus statements support a shift toward early, active, and individualized SRC management, reinforcing the role of athletic trainers in initiating symptom-limited activity and coordinating domain-specific rehabilitation.

Despite increasing convergence across consensus statements toward early, active, and multidomain concussion management, important differences remain in the specificity and operationalization of these recommendations. Earlier guidelines emphasized symptom resolution and rest, whereas more recent statements provide clearer direction regarding early aerobic exercise, targeted rehabilitation, and return-to-learn integration [2,4]. However, translation of these recommendations into clinical practice remains inconsistent. Implementation studies and survey data indicate that, although awareness of updated guidelines is high, adoption of key components such as vestibular screening, cervical assessment, and structured return-to-learn protocols varies widely across athletic training settings [3,9]. Barriers to implementation include limited access to specialized rehabilitation services, variability in clinician training and experience, and institutional or organizational constraints [3,9,10]. These findings highlight a persistent gap between consensus recommendations and real-world practice, underscoring the need for improved dissemination strategies, clinician education, and system-level support to facilitate consistent application of evidence-based concussion management.

### 3.3. Aerobic Exercise and Autonomic Regulation

Aerobic exercise emerged as one of the most extensively studied rehabilitation interventions in SRC management. Early studies published between 2000 and 2009 largely examined rest-based recovery, with observational data suggesting prolonged symptom duration among athletes prescribed strict inactivity [24,25]. Beginning around 2010, controlled studies evaluated subsymptom threshold aerobic exercise. These studies frequently used graded treadmill or cycling protocols guided by symptom exacerbation thresholds [6,26]. Across multiple randomized and prospective studies, initiation of subsymptom aerobic exercise within 7–14 days postinjury was associated with shorter recovery times, reducing median recovery by approximately 4–10 days. Exercise groups typically recovered in 13–15 days, compared with 17–24 days in rest or stretching-only control conditions [6,27,28]. Improvements were also observed in symptom severity scores, exercise tolerance, and autonomic function measures [6,27,28]. Importantly, no studies reported increased adverse events when exercise was prescribed below symptom thresholds. Findings were consistent across adolescent, collegiate, and adult recreational athlete populations. These findings are consistent with the use of early, symptom-limited aerobic exercise as part of contemporary SRC management, given its association with reduced symptom burden and shorter recovery timelines.

### 3.4. Vestibular and Oculomotor Rehabilitation

Vestibular and oculomotor impairments were commonly identified following SRC, particularly among athletes reporting dizziness, blurred vision, balance problems, and visual motion sensitivity [29,30]. Across observational and cohort studies, vestibular or oculomotor deficits were identified in approximately 30–60% of athletes during the subacute phase following SRC [29,30,31,32]. Among athletes with identified vestibular impairments, those receiving targeted vestibular rehabilitation achieved medical clearance 7–14 days earlier than those managed with usual care. Clearance rates of approximately 60–75% within 8 weeks were reported, compared with less than 20% in rest-based cohorts [31,32]. Targeted vestibular rehabilitation programs typically consisted of gaze stabilization, balance training, and habituation exercises delivered over 4–8 weeks. When positional vertigo suggestive of benign paroxysmal positional vertigo is identified, canalith repositioning maneuvers are indicated. These interventions may be performed prior to or alongside vestibular rehabilitation. However, routine BPPV screening was not a required component of the rehabilitation protocols in the cited studies. These programs were associated with clinically meaningful improvements in dizziness severity, balance performance, and functional mobility [31,33,34]. Several prospective studies reported that athletes receiving vestibular rehabilitation achieved medical clearance 7–14 days earlier than those managed without targeted therapy [35,36,37]. Pediatric-focused investigations demonstrated similar trends, with reductions in dizziness scores and gait instability observed after structured vestibular intervention, particularly in patients with persistent symptoms beyond 10–14 days postinjury [31,38,39]. These findings highlight the clinical importance of routine vestibular-ocular screening and support early referral for targeted vestibular rehabilitation to reduce symptom duration and expedite medical clearance. Coordination with vestibular specialists, physical therapists, or neuro-optometric providers may be necessary to facilitate timely intervention and optimize rehabilitation outcomes [31,33,34,35,37,39].

### 3.5. Cervical Spine Assessment and Rehabilitation

Cervical spine dysfunction emerged as a significant contributor to post-concussion symptomatology, particularly headache, neck pain, and dizziness. Studies published after 2014 reported cervical tenderness, reduced range of motion, or cervicogenic dizziness in 25–40% of athletes with SRC. Prevalence was higher among athletes experiencing prolonged recovery. Athletes reporting concurrent neck pain or cervical dysfunction were 1.5–2.5 times more likely to experience prolonged recovery beyond 28 days compared with those without cervical involvement [40,41,42]. Controlled and cohort studies evaluating cervical interventions, including manual therapy, mobility restoration, and strengthening, demonstrated increased likelihood of medical clearance by approximately 1.7–2.0 times within 6–8 weeks when incorporated into multimodal rehabilitation, compared with rest-based management [35,43,44,45,46,47]. These findings support routine cervical spine screening as part of comprehensive concussion evaluation and suggest that targeted cervical rehabilitation can meaningfully reduce recovery duration, particularly in athletes presenting with headache, neck pain, or dizziness. These findings also reinforce the importance of coordinated referral to clinicians trained in cervical manual therapy and rehabilitation when cervical involvement is identified [35,43,44].

### 3.6. Multimodal and Profile-Based Rehabilitation

An increasing number of studies examined rehabilitation strategies integrating multiple domains of care. In these programs, combined-domain care was associated with higher rates of symptom resolution by 6–8 weeks. Reported clearance rates ranged from 70 to 85%, compared with 40–55% in single-modality or rest-only management. Multimodal programs combining aerobic exercise, vestibular therapy, and cervical spine rehabilitation was associated with superior outcomes, including earlier symptom resolution and RTP clearance, compared with single-modality or rest-based care [43,48,49]. Reported differences ranged from approximately 10–21 days, particularly among athletes with mixed vestibular, cervical, and autonomic profiles [43,48,49,50]. Profile-based management strategies categorized athletes based on dominant symptom clusters. This framework supported individualized care planning and improved clinical efficiency [35,47,50]. These approaches were particularly effective in reducing prolonged symptoms among athletes with mixed vestibular, cervical, and autonomic profiles. Taken together, these findings support a multimodal, profile-based approach to SRC management, emphasizing coordinated intervention across impairment domains to optimize recovery and return-to-play outcomes.

### 3.7. Return-to-Learn Strategies

Research examining RTL expanded notably after 2015, particularly in adolescent and secondary school populations. In secondary school and collegiate samples, students implementing formal RTL accommodations demonstrated lower symptom burden at 2 weeks and fewer missed academic days compared with peers returning without structured support. Studies consistently reported that 40–50% of students experienced symptom exacerbation with full academic demands during early recovery [51,52,53]. Implementation of structured RTL protocols included reduced cognitive load, shortened school days, and temporary academic accommodations. These strategies were associated with improved symptom control and smoother school reintegration [54,55,56]. Schools utilizing formal RTL pathways demonstrated fewer reports of prolonged symptoms and reduced academic disruption compared with institutions lacking standardized RTL guidance. Evidence further suggested that earlier, supported return to school did not delay recovery and may mitigate psychosocial stressors associated with isolation and academic pressure [56,57,58]. These findings support early, structured return-to-learn implementation as a core component of SRC management, highlighting the role of athletic trainers in coordinating academic accommodations to minimize symptom exacerbation and psychosocial stress, although much of the current evidence is derived from observational and institutional studies.

### 3.8. Psychological and Behavioral Health Considerations

Psychological symptoms following SRC were frequently reported, with anxiety, depression, and sleep disturbance observed in 20% to 40% of athletes during recovery. Athletes with prolonged symptoms demonstrated higher rates of mood disturbance and stress-related complaints [59,60,61]. Observational studies examining integrated mental health screening and referral pathways reported associations with improved symptom trajectories and reduced duration of persistent post-concussive symptoms [2,62,63]. Although randomized trials in this domain remain limited, the consistency of observational findings supported inclusion of psychological assessment and behavioral health referral within multifaceted concussion management models [2,64,65]. These findings underscore the clinical value of routine psychological symptom screening and timely referral, supporting inclusion of mental health considerations within a comprehensive, multifaceted SRC management model, although current evidence in this domain remains primarily observational and heterogeneous.

### 3.9. Implementation and Practice Patterns

Despite growing evidence supporting multifaceted SRC management, implementation studies revealed persistent variability across athletic training settings, highlighting a continued gap between evidence-based recommendations and real-world clinical practice. Surveys conducted among athletic trainers indicated increasing adoption of multidomain assessment tools and active rehabilitation strategies; however, utilization of vestibular rehabilitation, cervical interventions, and formal RTL protocols remained inconsistent. Survey data indicate that more than 70% of athletic trainers report using multidomain concussion assessment batteries. However, only approximately 40–60% consistently implement vestibular or cervical screening. Fewer than 45–50% report routine use of formal RTL protocols [3,57,66,67,68]. Reported barriers included limited access to specialized services, variable clinician training, and institutional policy constraints [9,10,67]. These studies primarily relied on self-reported practice patterns and institutional surveys. They underscore the need for improved training, access to specialty services, and institutional support to facilitate consistent adoption of multifaceted SRC management strategies, although evidence describing implementation patterns remains heterogeneous across institutions and practice settings.

### 3.10. Clinical Relevance and Translational Implications

Collectively, the findings across rehabilitation domains inform key athletic training decision points. These include screening, referral, activity progression, and care coordination following SRC [3,9,65]. Early symptom-limited aerobic exercise supports assessment of exercise intolerance and graded physiologic progression [6,24,27,50]. Vestibular, oculomotor, and cervical findings further reinforce the importance of multidomain screening [7,31,35,69,70]. These assessments help guide targeted referral to domain-specific rehabilitation [31,35,43,47,65]. Return-to-learn and psychological considerations extend clinical management beyond physical recovery [22,51,52,53,58,59,62]. These domains highlight the need to monitor cognitive tolerance and coordinate interdisciplinary support [22,52,53,55,56,57,62]. Taken together, the evidence supports a coordinated, profile-informed model of care [2,9,65,66,70,71]. Within this model, athletic trainers integrate multidomain findings to guide rehabilitation progression and return-to-play decision-making [3,9,50,57,65,67].

## 4. Discussion

Over the past 25 years, sport-related concussion management has undergone a measurable conceptual and clinical transformation, shifting from prolonged rest toward active, impairment-driven rehabilitation [2,4]. Evidence across aerobic, vestibular–oculomotor, cervical, psychological, and academic domains demonstrates that recovery trajectories are influenced by interacting physiological and psychosocial systems [5,6,7,8]. Evidence from randomized trials, cohort research, and consensus recommendations supports modern concussion management. Care is now delivered through a coordinated, multifaceted model rather than rest alone [2,4]. This model integrates multidomain assessment, multimodal rehabilitation, and collaboration among providers. Athletic trainers are central in coordinating assessment, referral, and recovery progression [3,9]. Importantly, the maturity of evidence across SRC management domains remains uneven. Aerobic exercise interventions are supported by multiple randomized controlled trials, whereas evidence supporting vestibular rehabilitation, cervical treatment, psychological support, and return-to-learn strategies is derived more commonly from observational studies, prospective cohorts, and evolving clinical frameworks [2,4].

Within this review, terminology is used deliberately to clarify the structural hierarchy of contemporary SRC care. ‘Multifaceted’ refers to the overarching umbrella model recognizing concussion as a condition requiring integrated management across physiological, cognitive, and psychosocial systems. ‘Multidomain’ describes the clinical assessment structure used to evaluate impairment across discrete functional domains. ‘Multimodal’ refers to the coordinated delivery of multiple rehabilitation interventions. ‘Profile-based’ describes the clinical categorization of patients according to dominant symptom and impairment patterns to guide targeted management.

### 4.1. Paradigm Shift Toward Active Rehabilitation

**Figure 2:**
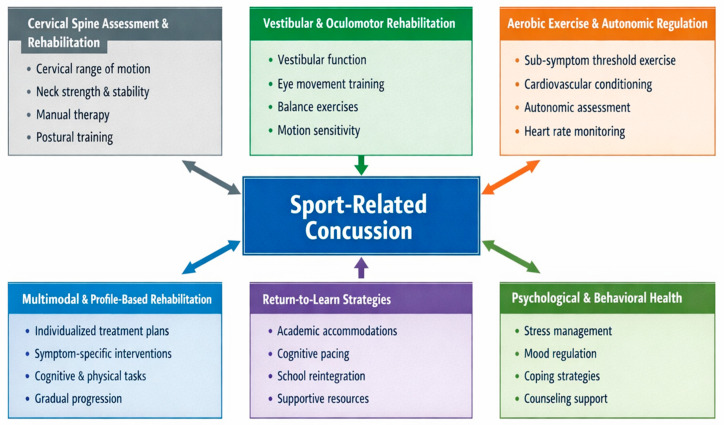
Conceptual Model of Multifaceted Sport-Related Concussion ManagementContemporary SRC care integrates multiple rehabilitation domains, including cervical spine assessment and rehabilitation, vestibular and oculomotor therapy, aerobic exercise and autonomic regulation, psychological and behavioral health support, return-to-learn strategies, and multimodal profile-based rehabilitation. These domains interact to guide individualized clinical management, symptom resolution, and return-to-activity outcomes.

This narrative review synthesizes 25 years of evidence in SRC management. The evidence demonstrates a sustained shift from prolonged rest-based paradigms toward active, multimodal rehabilitation within a broader multifaceted care framework [2,4,19,21]. Across rehabilitation domains, the available literature collectively suggests that early targeted intervention is associated with reduced symptom burden and faster clinical recovery compared with strict inactivity [6,25,35]. In the contemporary literature, this shift is reflected in evolving international consensus recommendations. It is also supported by randomized and prospective evidence showing that recovery is influenced by interacting autonomic, vestibular–ocular, cervical, cognitive, and psychological systems [2,5,59]. Collectively, the evidence supports a revised care model. In this model, athletic trainers initiate symptom-limited activity early and identify domain-specific impairments. This approach emphasizes coordinated rehabilitation and referral rather than reliance on rest until complete symptom resolution [2,3,4,9]. A conceptual framework illustrating the multifaceted care model of contemporary sport-related concussion management, including its multidomain assessment structure, is presented in Figure 2.

### 4.2. Domain-Specific Rehabilitation Implications

Aerobic exercise represents one of the most robust and consistently supported interventions in modern concussion management. Early work challenged the long-held assumption that strict physical rest is essential for neurological recovery and suggested that prolonged inactivity may delay return to normal functioning [4,6,24,25]. Subsequent randomized and prospective trials provide converging evidence supporting subsymptom threshold aerobic exercise. When initiated within approximately 7–14 days postinjury, this approach is safe and accelerates recovery without increasing adverse events [6,26,27,50]. Across studies summarized in this review, median recovery times were approximately 4–10 days shorter with early aerobic exercise compared with rest -based protocols [6,27,28,50]. Exercise groups commonly recovered in 13–15 days, compared with 17–24 days in control conditions [6,27,28,50]. These findings directly support a practice shift that is highly actionable for athletic trainers. Management moves from symptom avoidance to symptom-guided activity progression as a foundational component of multimodal concussion rehabilitation.

### 4.3. Vestibular and Oculomotor Rehabilitation Implications

Evidence supporting vestibular and oculomotor rehabilitation further underscores the clinical value of domain-specific assessment and intervention. Vestibular or oculomotor impairments are commonly identified in the subacute period following concussion. Observational and cohort data report deficits in approximately 30–60% of athletes, particularly among those with dizziness, balance problems, blurred vision, and visual motion sensitivity [7,29,31,32,69]. Targeted vestibular rehabilitation programs, typically delivered over 4–8 weeks and incorporating gaze stabilization, balance training, and habituation, are associated with meaningful improvements in dizziness, balance, and functional mobility [31,33,34,35,36]. Athletes receiving vestibular rehabilitation achieved medical clearance approximately 7–14 days earlier than those managed without targeted therapy. Reported clearance rates were approximately 60–75% within 8 weeks, compared with less than 20% in usual care cohorts [31,35,36]. From a clinical standpoint, these findings reinforce the importance of routine vestibular–ocular screening as part of a multidomain concussion assessment within athletic training protocols. Failure to identify this impairment profile can delay access to targeted intervention [31,35]. This delay may prolong symptoms and recovery timelines.

### 4.4. Cervical Spine Contributions to Post-Concussion Symptoms

Cervical spine involvement represents another critical and historically underrecognized contributor to post-concussion symptoms. Cervical tenderness, restricted range of motion, and cervicogenic dizziness are frequently reported following concussion. Studies identified cervical-related findings in approximately 25–40% of athletes, with higher prevalence among those with prolonged recovery [35,41,69,72,73]. When cervical rehabilitation is incorporated into multimodal care, outcomes improve compared with rest based approaches. Multimodal programs that included cervical interventions demonstrated an increased likelihood of medical clearance compared with rest-based management. Reported estimates ranged from approximately 1.7–2.0 times higher within 6–8 weeks [35,74,75]. These findings support routine cervical screening as part of a multidomain concussion assessment. They also highlight the clinical risk of attributing headache, dizziness, or neck-related symptoms solely to central neurological mechanisms [35,41].

### 4.5. Multimodal Rehabilitation and Profile-Based Clinical Categorization

The literature on multimodal rehabilitation and profile-based clinical categorization further reinforces the limitations of single-modality care. Programs integrating aerobic exercise, vestibular therapy, and cervical spine rehabilitation have been associated with superior outcomes compared with rest-only or isolated interventions. These benefits are particularly evident among athletes presenting with mixed impairment profiles [70,76,77,78]. Multimodal management was associated with earlier symptom resolution and RTP clearance compared with rest-based management. Reported differences ranged from approximately 10–21 days. Profile based approaches that categorize athletes by dominant symptom clusters and functional deficits offer a practical framework for individualizing care, improving clinical efficiency, and guiding targeted referrals [9,70,71,75]. For athletic trainers, these findings support a care coordination role that integrates multidomain assessment with multimodal rehabilitation planning, progression, referral, and communication across the medical team, including physical therapists, vestibular specialists, physicians, and mental health providers when domain-specific impairments are identified.

### 4.6. Athletic Trainers as Coordinators of Multidomain Care

From a clinical process perspective, the evolution of SRC management over the past 25 years necessitates a fundamental shift in how athletic trainers approach evaluation and care delivery [2,3,9]. Earlier approaches emphasized prolonged rest, symptom monitoring in isolation, and delayed progression until complete symptom resolution [4,25]. Contemporary evidence instead supports early, active, and domain-specific assessment beginning in the acute and subacute phases of injury [6,24,27,50]. For athletic trainers, this shift requires routine multidomain screening across multiple systems, including exercise tolerance, vestibular–ocular function, cervical spine involvement, cognitive load, and psychological symptoms, rather than reliance on symptom checklists alone [7,41,69,70]. Clinical decision-making now centers on identifying dominant impairment profiles, initiating symptom-limited activity early, and coordinating targeted referrals when deficits are identified [35,45,47,65]. This approach reframes concussion management as an adaptive, multidimensional process rather than a linear rest-to-return progression, positioning athletic trainers as central coordinators of care throughout recovery [2,10,66].

### 4.7. Return-to-Learn and Academic Reintegration

RTL has emerged as an essential component of comprehensive SRC management, especially in adolescent populations where academic demands can meaningfully influence symptom experience. Across studies summarized in this review, approximately 40–50% of students experienced symptom exacerbation with full academic demands early in recovery [22,79,80,81,82]. Evidence indicates that structured return to learn protocols, including temporary workload reductions, modified schedules, and academic accommodations, support smoother reintegration and may reduce psychosocial stressors associated with isolation and academic pressure [22,80,82,83,84]. The literature summarized here suggests that earlier supported return to school does not delay recovery. These findings challenge earlier assumptions that cognitive rest should mirror physical rest [2,22,25,58]. These findings align with a broadened athletic training role that includes communication with educators, administrators, and families to facilitate safe and effective academic progression [22,57,67,80].

### 4.8. Psychological and Behavioral Health Integration

Psychological and behavioral health considerations further emphasize the multidimensional nature of concussion recovery. Anxiety, depression, and sleep disturbance were commonly reported during recovery. Estimates of psychological symptoms ranged from approximately 20% to 40% of athletes [19,77,85,86]. Athletes with prolonged symptoms also demonstrate higher rates of mood disturbance and stress related complaints, supporting a bidirectional relationship between concussion symptoms and mental health. Although randomized trials in this domain remain limited, observational evidence collectively supports routine mental health screening and timely referral pathways as components of multifaceted concussion care models [2,19,59,85,86]. For athletic trainers, integrating brief screening, symptom monitoring, and referral mechanisms can complement physiologic rehabilitation and may reduce the likelihood of persistent symptoms [6,35,50,71].

### 4.9. Implementation Gaps in Athletic Training Practice

Despite strong evidence supporting multifaceted SRC care models, implementation gaps in multidomain assessment and multimodal rehabilitation persist across athletic training settings. Survey data indicate that more than 70% of athletic trainers report using multidomain concussion assessment batteries. However, only approximately 40–60% consistently implement vestibular or cervical screening. Fewer than 50% report routine use of formal return-to-learn protocols [3,57,67,87]. Reported barriers include limited access to specialized services, variable clinician training, and institutional policy constraints [10,88]. These findings highlight a continuing gap between evidence-based recommendations and real world practice [3,57,67], and they suggest that dissemination of evidence alone is insufficient to ensure consistent implementation.

Structural factors may also contribute to variability in the implementation of multidomain concussion management. Differences in resource availability across athletic settings, including access to vestibular specialists, physical therapists, and mental health providers, may influence the feasibility of referral-based care models. Athletic trainers practicing in smaller institutions, rural settings, or resource-limited environments may face greater barriers to accessing specialized rehabilitation services. In addition, variability in clinician training and continuing education opportunities related to vestibular–ocular rehabilitation, cervical spine management, and psychological screening may influence confidence and adoption of these practices. Institutional policies, medico-legal considerations, and inconsistent integration of return-to-learn protocols within school systems may further complicate implementation of comprehensive concussion management strategies [3,57,67].

### 4.10. Strategies to Improve Clinical Implementation

Several studies and position statements have proposed strategies to help bridge this implementation gap [2,9,66]. Suggested approaches include expanding continuing education focused on vestibular, cervical, and psychological concussion domains; developing formal referral networks with vestibular specialists, mental health providers, and neurocognitive clinicians; and establishing standardized, institution-level concussion management protocols that incorporate return-to-learn and multidomain rehabilitation pathways [2,9,51,52,53,58,65,67]. These system-level supports may enhance clinician confidence, improve access to domain-specific care, and facilitate more consistent adoption of multidomain assessment and multimodal rehabilitation within evidence-based multifaceted concussion care models across athletic training settings [3,57,66,67].

### 4.11. Study Limitations

Several limitations should be considered when interpreting this review. Formal risk-of-bias scoring and quantitative pooling were not performed. Heterogeneity in study design, outcomes, and intervention protocols limits direct comparison across studies. However, this variability reflects the complexity inherent in real-world concussion management environments [15,16,18,89]. As a narrative review, study selection and thematic synthesis involve interpretive judgment, which may introduce emphasis bias. Publication bias must also be considered, as studies demonstrating positive rehabilitation outcomes are more likely to be published. Additionally, the review was limited to peer-reviewed literature indexed in selected databases and published in English. Despite these limitations, consistent findings across multiple populations and methodologies support the conclusion that early active rehabilitation and domain-specific interventions improve recovery outcomes compared with prolonged rest.

### 4.12. Future Research Directions

Future research should prioritize high-quality randomized trials evaluating integrated rehabilitation models, particularly in pediatric and high school populations where SRC burden and RTL needs are substantial. Additional work is needed to clarify the optimal timing, dosing, and sequencing of interventions across impairment profiles. Further research should determine which combinations of aerobic, vestibular–ocular, cervical, academic, and psychological interventions yield the greatest benefit for specific patient presentations. Equally important, implementation science studies are needed to identify scalable strategies that improve adoption of evidence-based multifaceted management in resource-limited settings. These strategies may include clinician training models, referral network development, and policy-level support.

## 5. Conclusions

Over the past 25 years, SRC management has shifted from prolonged rest toward an active, multifaceted model integrating multidomain assessment and multimodal rehabilitation. Evidence across aerobic exercise, vestibular–oculomotor therapy, cervical spine management, return-to-learn strategies, and psychological support has been associated with improved recovery trajectories compared with rest-based care. For athletic trainers, these findings support early symptom-limited activity, domain-specific intervention, and coordinated interprofessional management. Although the strength of evidence varies across rehabilitation domains, the collective literature supports a multifaceted approach to concussion care. Continued efforts to improve implementation and access are necessary to ensure consistent, evidence-based concussion management across athletic settings.

## Figures and Tables

**Table 1:** Summary of Evidence Supporting a Multifaceted Approach to Sport-Related Concussion Management (2000–2025).

Rehabilitation Domain	Key Interventions	Study Designs Represented	Populations Studied	Direction of Evidence	Clinical Implications for Athletic Trainers
Aerobic exercise and autonomic regulation	Sub-symptom threshold treadmill or cycling exercise; graded aerobic progression	Randomized controlled trials; prospective cohort studies; systematic reviews	Adolescent, collegiate athletes, adults	Consistent evidence supporting reduced symptom duration and faster recovery compared with rest-based care	Early, symptom-limited aerobic exercise can be safely prescribed to facilitate recovery and reduce prolonged symptoms
Vestibular and oculomotor rehabilitation	Gaze stabilization; balance training; habituation exercises; visual motion sensitivity training	Prospective cohorts; controlled trials; systematic reviews	Pediatric, adolescent, and collegiate athletes	Positive association with reduced dizziness, improved balance, and earlier medical clearance	Routine vestibular screening and targeted referral should be incorporated into concussion evaluation and management
Cervical spine assessment and rehabilitation	Manual therapy; mobility restoration; strengthening; postural correction	Cohort studies; controlled clinical trials; multimodal intervention studies	Adolescents and collegiate athletes	Evidence supports improved headache, neck pain, and dizziness outcomes when cervical dysfunction is addressed	Cervical screening should be included in concussion evaluations to identify treatable contributors to symptoms
Multimodal rehabilitation	Combined aerobic, vestibular, and cervical interventions; individualized care plans	Prospective cohorts; clinical trials; narrative and scoping reviews	Adolescent and collegiate athletes	Superior outcomes compared with single-modality or rest-only approaches	Athletic trainers should coordinate integrated rehabilitation strategies based on symptom profile
Profile-based concussion management	Symptom cluster classification (vestibular, cervicogenic, autonomic, cognitive, mood)	Cohort studies; conceptual frameworks	Adolescents and adults	Supports individualized treatment selection and improved clinical efficiency	Symptom profiling can guide targeted intervention and referral decisions
Return-to-learn strategies	Gradual cognitive reintegration; academic accommodations; reduced workload	Observational studies; cohort studies; guideline statements	Middle school, high school, and collegiate students	Early supported return-to-learn associated with improved symptom control and reduced academic disruption	Athletic trainers should collaborate with educators to implement structured return-to-learn plans
Psychological and behavioral health support	Mental health screening; counseling referral; sleep and stress management	Observational studies; cohort studies	Adolescents and collegiate athletes	Consistent association between psychological support and improved symptom trajectories	Incorporating mental health screening and referral may reduce risk of prolonged recovery
Implementation and practice patterns	Policy adoption; clinician education; access to multidisciplinary care	Survey studies; implementation research	Athletic trainers across sport settings	Variable uptake despite strong evidence base	Institutional support and education are necessary to translate evidence into practice

**Table 2:** Evolution of Sport-Related Concussion Management and Key Guideline Milestones (2000–2025).

Year	Organization or Guideline	Population Focus	Key Management Recommendations	Notable Shift From Prior Guidance
2001	Concussion in Sport Group (Vienna Consensus)	Adult and elite athletes	Emphasized symptom resolution and physical rest before return-to-play; limited guidance on rehabilitation or cognitive activity	Formalized concussion management while reinforcing a rest-based recovery paradigm
2004	National Athletic Trainers’ Association Position Statement	Secondary school and collegiate athletes	Recommended immediate removal from play, symptom monitoring, and graded return-to-play following symptom resolution	Established athletic trainer-led responsibility for concussion recognition and management
2008	Concussion in Sport Group (Zurich Consensus)	Youth and adult athletes	Continued emphasis on rest until asymptomatic; introduced stepwise return-to-play progression	Acknowledged variability in recovery timelines
2012	Concussion in Sport Group (Zurich Update)	Youth and adult athletes	Recognized potential drawbacks of prolonged strict rest; suggested gradual return to activity	First indication that excessive rest may negatively affect recovery
2014	National Athletic Trainers’ Association Position Statement Update	Athletic trainers across sport settings	Expanded guidance on assessment tools, documentation, and clinical decision-making	Strengthened clinical framework for athletic trainer-directed concussion care
2015	American Academy of Pediatrics Clinical Report	Pediatric and adolescent athletes	Emphasized cognitive rest with gradual return-to-learn and early academic accommodations	Elevated return-to-learn as a core component of concussion recovery
2016	Concussion in Sport Group (Berlin Consensus)	Youth, collegiate, and elite athletes	Recommended brief rest (24–48 h) followed by symptom-limited physical activity	Explicit shift away from prolonged rest toward early activity
2018	American Academy of Pediatrics Policy Statement Update	Pediatric and adolescent populations	Supported early return to school with academic supports; discouraged prolonged school absence	Reinforced return-to-learn as essential to recovery
2019	Rehabilitation literature (cervicovestibular models)	Adolescent and collegiate athletes	Supported targeted vestibular, oculomotor, and cervical rehabilitation for persistent symptoms	Advanced multidomain, profile-based rehabilitation concepts
2022	Concussion in Sport Group (Amsterdam Consensus)	Youth and adult athletes	Endorsed early aerobic exercise, targeted rehabilitation, and individualized management strategies	Formalized active rehabilitation and multidomain care as best practice
2023	Amsterdam Consensus Publications	Broad sport populations	Introduced updated assessment tools and refined return-to-play and return-to-learn guidance	Integrated assessment, rehabilitation, and recovery monitoring
2024	National Athletic Trainers’ Association Bridge Statement	Athletic trainers across clinical settings	Translated Amsterdam consensus recommendations into athletic trainer-specific guidance	Bridged international consensus science to real-world athletic training practice
2025	Emerging clinical and implementation research	Secondary school and collegiate settings	Emphasized multidisciplinary care, mental health screening, and implementation strategies	Shifted focus toward improving uptake and consistency of multifaceted concussion care

## Data Availability

No new data were created or analyzed in this study. Data sharing is not applicable to this article.

## Abbreviations

The following abbreviations are used in this manuscript:

SRC	Sport-Related Concussion
RTP	Return-to-Play
RTL	Return-to-Learn
mTBI	Mild Traumatic Brain Injury
NATA	National Athletic Trainers’ Association
CISG	Concussion in Sport Group
AAP	American Academy of Pediatrics
BPPV	Benign Paroxysmal Positional Vertigo
